# Investigating Awareness Regarding Travel-Related Infectious Disease Prevention in a Metropolitan Area

**DOI:** 10.3390/tropicalmed8100476

**Published:** 2023-10-18

**Authors:** Francesca Pennino, Claudio Fiorilla, Michele Sorrentino, Umberto Armonia, Antonio Parisi, Pasquale Domenico Mirizzi, Maddalena Di Lillo, Ornella De Silva, Paolo Montuori, Maria Triassi, Antonio Nardone

**Affiliations:** Department of Public Health, “Federico II” University, Via Sergio Pansini No. 5, 80131 Naples, Italy

**Keywords:** travel, infectious diseases, knowledge, attitude, practice, cross-sectional survey

## Abstract

The rise in international travel has led to an increase in travel-related infectious diseases. It is predicted that by 2030, the number of international travelers will reach 1.8 billion, with over 250 million people affected globally. This issue also has an economic impact, as the eradication of travel-related infectious diseases leads to a loss of USD 12 billion in tourism. To understand the association between demographic variables and knowledge, attitude, and behaviors related to travel-related infectious diseases, a cross-sectional survey-based study was conducted among 1191 individuals in the metropolitan city of Naples, Italy. Multiple linear regression was performed over three models. The results revealed that knowledge about travel-related infectious diseases was positively associated with age, female gender, non-smoking habits, being single, and higher education attainment. The attitude towards travel-related infectious diseases was positively associated with being female, non-smoking habits, being single, higher education attainment, and a higher level of knowledge. A statistically significant association was observed between behaviors and non-smoking habits and between higher levels of knowledge and attitudes. To address this issue, public health programs could be implemented to improve behaviors in the general population. Overall, this study provides valuable information about the determinants of knowledge, attitude, and behaviors related to travel-related infectious diseases in the general population.

## 1. Introduction

Travel-related infectious diseases are a major concern in the modern era [1], due to the increase in international travel, which is predicted to return to pre-pandemic levels [2] and is estimated to reach 1.8 billion by 2030 [3]. This increase in travel has contributed to the amplification of infectious diseases [4,5,6], with global outbreaks recently documented [7,8,9,10,11]. Recently, over 250 million individuals have experienced a travel-related infection worldwide, with a death toll of over 650,000 [12,13,14]. In Italy, the escalating number of travelers, notably facilitated by Naples Airport’s status as the largest gateway in southern Italy, has contributed to the rise in international travel [15]. This trend has significantly contributed to the persistence of malaria as the primary imported infectious disease in non-endemic countries [16]. In fact, around 20% of malaria cases during the period 2000–2016 have been reported in travelers [17]. Furthermore, instances of dengue and chikungunya cases were associated with international travel to Italy [18]. Nonetheless, research conducted in Italy has highlighted a significant knowledge gap regarding travel-related infectious diseases [19], emphasizing the need for increased attention and improvement in this area. Travel-related infectious diseases also impact the economic burden; the potential savings from eradicating these diseases are estimated at USD 12 billion in tourism expenditures [20]. These diseases, characterized by their intricate and opportunistic spread, pose a considerable challenge to public health efforts [21]. It is widely acknowledged that travel remains a key factor in the transmission of infectious diseases [22,23,24], although more individuals express hesitancy to visit countries with a high disease prevalence [25]. However, despite this hesitancy, trips to such areas continue to grow [5], resulting in up to 87% of travelers contracting illnesses upon return [26]. The likelihood of contracting a travel-related infectious disease is determined not only by the destination but also by the individual’s personal risk profile [27], which is influenced by many variables such as age, gender, culture, social status, and education attainment [28]. Despite the availability of preventive measures, such as vaccinations and pre-travel consultations [28], many travelers tend to underestimate the risk of contracting infectious diseases, indicating a general lack of awareness regarding health risks [19,29,30,31] and preventive measures [19,32]. Therefore, it is imperative to improve counseling and education about infectious diseases related to travel, as well as preventive measures [33,34].

Several studies in the literature have investigated knowledge, attitudes, and behaviors towards travel-related infectious diseases; however, these studies mainly focus on specific populations, such as international travelers or frequent flyers, or on a specific travel-related infectious disease [27,28,35,36,37,38,39,40,41,42,43,44,45,46,47,48]. The literature highlights the significant need for education, as travelers often lack awareness of potential health hazards and fail to take essential precautions, such as getting vaccinated or seeking medical advice prior to their travels. Some studies have specifically targeted healthcare workers [48,49], demonstrating the need for interventions to improve their knowledge and enable them to play an active role in counseling the public and improving the management of travelers.

Analyzing knowledge and attitudes towards travel-related infectious diseases in a broader population might help identify gaps in knowledge that may affect decision-making and behavior, thus mitigating the risk of transmitting infectious diseases. By recognizing potential barriers and enablers to adopting preventive measures and implementing appropriate policies, those measures might enhance overall health outcomes. However, there is a significant gap in the existing literature, with only one study [19] conducted among the general population in a metropolitan area regarding travel-related infectious diseases. Therefore, to delve into the interplay between demographic variables and knowledge, attitudes, and behaviors concerning travel-related infectious diseases, a cross-sectional survey-based study was conducted within the metropolitan city of Naples, Italy. The primary aim of this study is to address critical knowledge gaps and implement efficient measures for controlling and reducing the transmission of infectious diseases during travel.

## 2. Materials and Methods

### 2.1. Setting and Sample 

The present study employed a cross-sectional design to examine the knowledge, attitudes, and behaviors related to travel-related infectious diseases among adult participants in the metropolitan city of Naples, Italy. This study was conducted from December 2022 to March 2023, and questionnaires were administered to 1523 subjects. The initial participants were identified and recruited using a snowball sampling method, starting with a trusted trial group recommending 2–3 subjects who represented a diverse selection from the general population within the metropolitan area and had expressed interest in participating in our study. Subsequently, we reached out to potential participants through multiple channels, including community organizations, local health clinics, and social media platforms, to ensure a varied and representative sample and reduce the selection bias. The final sample included 1191 participants, and the response rate was 78.29%. To meet the requirements for inclusion, participants needed to be eligible by being aged 18 and above and also residing within the metropolitan area of Naples. Some characteristics were taken into account during sampling to increase the representativeness of the sample, such as gender distribution, education, marital status, and age [50].

The sample size was determined using Slovin’s formula with the objective of achieving a margin of error of 3% and a confidence interval of 95%. The estimated number of participants was 1523. However, accounting for a non-response rate of 30%, the final estimated sample size was 1066. 

### 2.2. Procedures 

Experienced interviewers were responsible for administering the questionnaire to participants throughout the study period. The data collection occurred from Monday to Friday, specifically between 10:00 a.m. and 8:00 p.m. This time frame was chosen to avoid overrepresentation of unemployed individuals or students. The interviewers clearly identified themselves as representatives of the University of the Studies of Naples “Federico II” and provided comprehensive information to the participants regarding the nature and scope of our research, the methodology employed, and the voluntary nature of participation. Participants were assured that all collected data would be treated with strict anonymity and confidentiality, and they were informed of their right to withdraw from our study at any time without providing a reason. Prior to the commencement of the interview, participants were required to provide consent. It is important to note that no incentives or rewards were provided to participants for their involvement in or completion of the survey. Throughout this study, strict adherence to the guidelines and principles outlined in the Declaration of Helsinki was maintained, and ethical clearance was obtained according to local legislation.

### 2.3. Data Collection 

The questionnaire utilized in this study was developed by a substantial committee of healthcare professionals, including physicians and other healthcare workers. To ensure the questionnaire’s relevance and appropriateness to the study’s objectives, any questions that were deemed unsuitable or unrelated were either substituted or eliminated. Before the commencement of data collection, a pilot study was conducted involving 10 individuals to assess their comprehension of the questionnaire items. It should be noted that the results from the pilot study were not considered in the final analysis of the main study.

The questionnaire was divided into two sections. The first section gathered socio-demographic data and other health-related information, such as gender, age, marital status, having children, education level, and smoking habits. In the second section, participants were presented with a total of 30 questions that assessed their knowledge, attitudes, and behaviors concerning travel-related infectious diseases. The questions pertaining to knowledge and attitudes utilized a three-point Likert scale, offering response options of “agree”, “uncertain”, and “disagree”, which were coded as 1, 2, and 3, respectively. The behavior-related questions offered four response options: “never”, “sometimes”, “often”, and “yes/always”, which were coded as 1, 2, 3, and 4, respectively.

### 2.4. Statistical Analysis 

The data collected in this study were analyzed using the STATA MP v14.0 statistical software program (College Station, TX, USA). The analysis was conducted in two stages. In the first stage, descriptive statistics were used to summarize the basic information of the statistical units. In the second stage, a multiple linear regression analysis (MLRA) was performed over three models: Model I, Model II (partially adjusted), and Model III (fully adjusted). The dependent variables (knowledge, attitudes, and behaviors) were calculated by adding the scores obtained from the corresponding questions (questions with inverse answers were coded inversely). The independent variables were included in all models and consisted of sex (1 = male, 2 = female), age in years, education level (1 = primary school, 2 = middle school, 3 = high school, 4 = university degree), marital status (1 = single, 2 = in a relationship), smoking habits (1 = smoker, 2 = non-smoker), and having children (1 = yes, 2 = no). In Model II, knowledge was added to the independent variables, and in Model III, knowledge and attitudes were added to the independent variables. All statistical tests were two-tailed, and results were considered statistically significant if the *p*-values were less than or equal to 0.05.

## 3. Results

The characteristics of the study population are presented in Table 1. The sample consisted of 46% males and 54% females, with a mean age of 45.84 years and a range of 18–84 years (S.D. ± 16.24). The majority of participants were over 51 years old (40.4%), followed by those under 30 years old (21.9%). Approximately half of the participants did not have children (48.1%), and the majority of them were in a relationship (58.4%). Moreover, 56% of the respondents reported not smoking.

Table S1 displays the results of this study’s investigation into the participants’ knowledge of travel-related infectious diseases. While 77% of the respondents correctly identified that bacterial/viral/fungal entry into the body causes infections, only 31.40% believed that a body temperature exceeding 38 °C constitutes a fever. Our study also found that 40.55% of the participants knew that contaminated water is the primary mode of infection transmission, while 48.24% were aware that mosquitoes can transmit diseases. Furthermore, 44.92% of the respondents correctly knew that vaccines stimulate antibody production. Interestingly, 25.10% of the respondents mistakenly assumed that medical assistance is free worldwide. Lastly, only 25.9% of the respondents were aware that they can get vaccinated before traveling at Maritime, Air, and Border Health Offices (USMAFs).

Table S2 displays the participants’ attitudes toward travel-related infectious diseases. The results indicate that 35.43% of the respondents believe that packing drugs for travel is unnecessary. A significant proportion of respondents believe that disinfecting is always necessary after sustaining a cut (44.67%), while 22.75% believe that covering mouths when sneezing or coughing is unnecessary. This study also underscores the divergence of perspectives concerning tap water consumption while traveling: 30.98% express agreement, while 32.66% remain uncertain. In contrast, 36.36% firmly disagree. Additionally, 26.95% of the participants agree that eating with their hands is enjoyable, whereas 40.47% disagree. Our study also found that 36.94% of the participants believe that wearing a mask when using public transportation is unnecessary, while 46.68% think it is important to carry sanitizer. Furthermore, 29.63% of the respondents believe that medical insurance is pointless while traveling, but 43.24% consider it useful to research necessary vaccines before traveling. Table S3 presents the distribution of responses regarding behaviors related to travel-related infectious diseases. The results reveal that 30.73% always travel abroad, while 28.30% always carry medications. A total of 29.22% of the respondents stated that they frequently consume local cuisine, even if it is not cooked. Only 27.62% of the participants reported that they exclusively drink bottled water while abroad, and 29.47% admitted to often eating street food abroad. Merely 32.16% of the respondents stated that they always inquire about the need for vaccines before traveling, and 36.86% reported using anti-mosquito products such as sprays, diffusers, or bracelets. Lastly, 37.28% of the respondents reported having medical insurance when traveling abroad. 

Table 2 presents the results of the multiple logistic regression analysis (MLRA) in three models.

**Figure 1:**
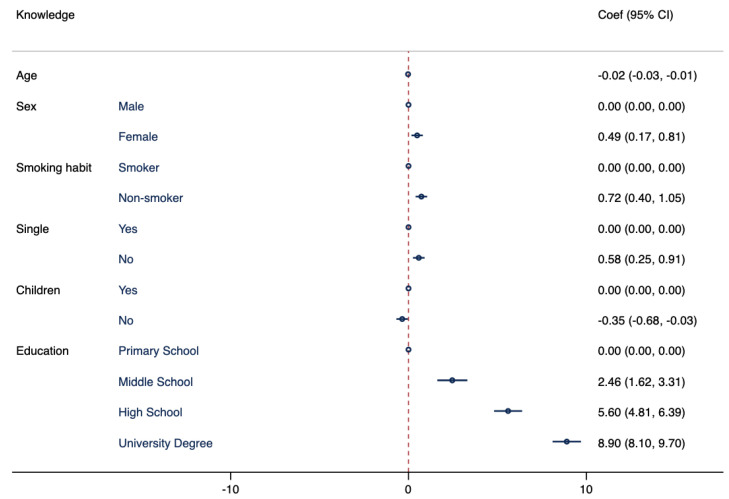
Association between knowledge regarding travel-related infectious diseases and demographic characteristics. Multivariate logistic regressions were employed, including knowledge regarding travel-related infectious diseases as an outcome variable and controlling for the following variables: age, sex, smoking habits, marital status, having children, and education attainment. Results are presented as Coef and CI.

Model I indicates an association between knowledge regarding travel-related infectious diseases and age, gender, smoking habits, marital status, and education level, as shown in Figure 1.

**Figure 2:**
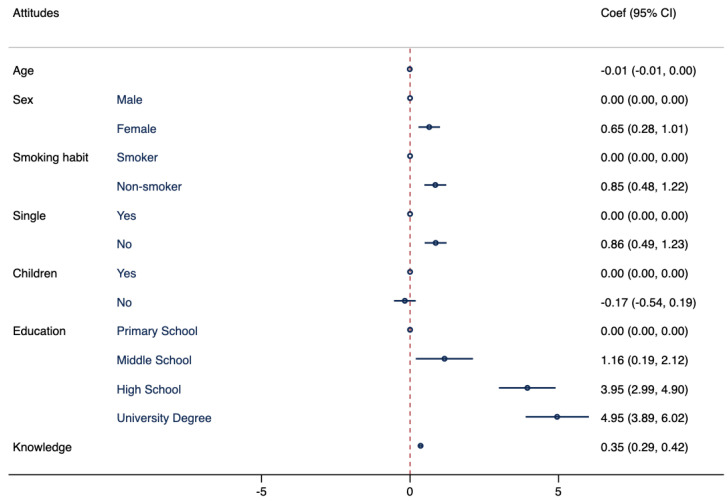
Association between attitude regarding travel-related infectious diseases and demographic characteristics. Multivariate logistic regressions were employed, including attitude regarding travel-related infectious diseases as an outcome variable and controlling for the following variables: age, sex, smoking habits, marital status, having children, education attainment, and knowledge. Results are presented as Coef and CI.

Model II shows a statistically significant association between attitudes toward travel-related infectious diseases and gender, smoking habits, education level, marital status, and knowledge, as illustrated in Figure 2. 

**Figure 3:**
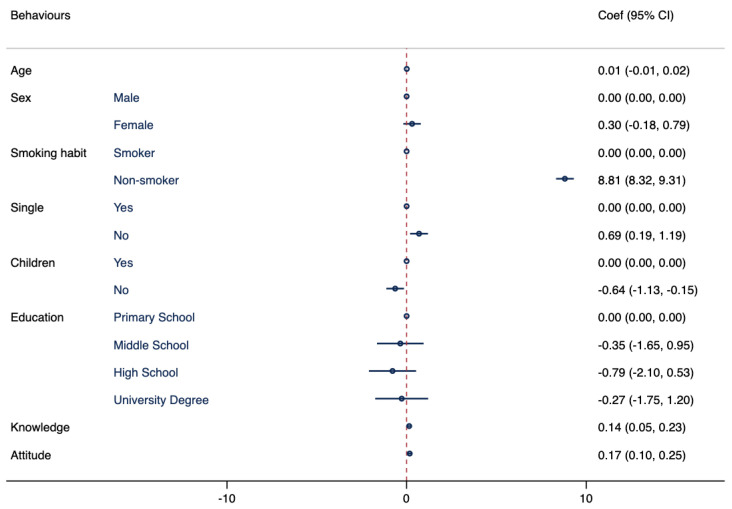
Association between behaviors regarding travel-related infectious diseases and demographic characteristics. Multivariate logistic regressions were employed, including behaviors regarding travel-related infectious diseases as an outcome variable and controlling for the following variables: age, sex, smoking habits, marital status, having children, education attainment, knowledge, and attitude. Results are presented as Coef and CI.

In Model III, as shown in Figure 3, a statistically significant association is observed between behaviors and marital status, smoking habits, knowledge, and attitudes. 

## 4. Discussion

This study emphasizes the significance of understanding travel-related infectious diseases in the context of today’s globalized world to effectively prevent the rapid spread of disease outbreaks. It explores the intricate relationship between demographic factors and the knowledge, attitudes, and behaviors of individuals regarding travel-related infectious diseases within a major urban area. Model I illustrates a noteworthy association between younger age and knowledge of travel-related infectious diseases. This finding aligns with a previous study conducted among Chinese travelers [44] but contradicts the results of studies conducted among Arabian [45] and Egyptian [41] travelers. These differences might be the result of socio-cultural disparities, where variations in education, healthcare access, and cultural norms regarding travel and health can substantially impact knowledge levels concerning travel-related infectious diseases. However, no differences were found among Dutch travelers [36]. It is plausible to suggest that younger individuals possess greater knowledge about travel-related infectious diseases due to their higher level of familiarity with computers and the Internet, which serve as the most common sources of pre-travel advice [43]. 

Our study found an association between being female and higher knowledge about infectious diseases related to travel. This finding aligns with studies conducted on Chinese travelers [44] but contrasts with a study conducted on Omani travelers [28], where a larger proportion of male participants (72.5%) was involved. This distinction may be attributed to the setting and context of the study. Additionally, the variation in results could be attributed to the fact that women tend to be more cautious about their health [51], which may prompt them to actively seek greater amounts of travel advice. The observed association between non-smoking habits and knowledge about travel-related infectious diseases highlights a significant aspect of public health. Individuals who refrain from smoking demonstrate a greater awareness and understanding of health risks, including those associated with infectious diseases encountered during travel [52,53]. This finding emphasizes the potential benefits of promoting non-smoking behaviors and calls for further research to better understand the underlying mechanisms and investigate the potential causality between non-smoking habits and knowledge about travel-related infectious diseases. Our research has revealed an additional unique and previously unreported finding, namely, that being single is associated with having better knowledge about travel-related infectious diseases. Moreover, single individuals demonstrate higher health literacy compared to married individuals [53,54]. These findings suggest that single individuals may have more opportunities and flexibility to engage in health education and seek information, as they often have greater autonomy over their time and decision-making. To fully comprehend the implications of these associations, further investigation is necessary to assess their significance and explore the underlying factors that contribute to these patterns. Furthermore, our study supports previous research demonstrating a positive association between educational attainment and knowledge about travel-related infectious diseases. Several previous studies conducted in Korea, Egypt, and the USA [29,41,48] indicate that college graduates tend to have a higher level of knowledge compared to those with less education. This might be attributed to factors such as better health literacy, increased exposure to scientific information, and access to resources like peer-reviewed journals and academic publications. Conversely, individuals from disadvantaged socioeconomic backgrounds may face limited access to education [55,56]. In Model II, the first evidence indicates an association between being female and holding specific attitudes towards infectious diseases related to travel. This finding is unique in the literature and can be attributed to the tendency of females to take fewer risks and prioritize their health [51].

Furthermore, our study revealed a significant association between being single and both non-smoking habits and attitudes. These findings make novel contributions to the existing body of research in this area. Notably, the smoking habit could potentially serve as a proxy indicator for lower adherence to preventive measures. Current smokers demonstrated less inclination to utilize specific preventive services compared to non-smokers [57,58,59]. Similarly, being single emerged as an important predictor of healthcare utilization [60]. However, to fully comprehend the underlying mechanisms driving these associations, additional investigation is warranted. Further research will help shed light on the complex dynamics at play and provide a more comprehensive understanding of these relationships. Moreover, research consistently shows a positive association between educational attainment and attitudes regarding travel-related infectious diseases. This association aligns with the findings of the study conducted by Alghamdi and colleagues [45], which specifically focused on Arabian travelers. Our study further supports the idea that individuals with higher educational levels tend to exhibit more informed and proactive attitudes when it comes to travel-related infectious diseases. Additionally, this study demonstrates a noteworthy link between knowledge and attitude, suggesting that having knowledge about travel-related infectious diseases could contribute to the development of positive attitudes. This finding is consistent with previous research conducted among Arabian travelers [45] and Italian pharmacists [61], emphasizing the importance of accurate knowledge in fostering positive attitudes. The significance of this relationship is particularly relevant to public health promotion and the prevention of travel-related infectious diseases. Therefore, having access to reliable and precise information is crucial for making informed decisions and cultivating positive attitudes. 

The findings from Model III demonstrate a significant association between higher levels of knowledge and positive attitudes towards healthy behaviors.

This outcome aligns with earlier research conducted among both Egyptian [41] and Arabian travelers [45], indicating that having access to precise and dependable information regarding travel-related infectious diseases can encourage the adoption of behaviors. Furthermore, our study uncovered a unique and distinct association between non-smoking habits and behaviors, which is not commonly observed in the existing literature. This novel finding adds a valuable contribution to the understanding of how smoking habits may relate to various behavioral aspects, particularly considering how smoking is often associated with other unhealthy behaviors [62,63]. By highlighting this connection, our research emphasizes the importance of considering the broader context of smoking behavior and its potential implications for other health-related behaviors. Further exploration of this association is warranted to enhance our understanding of the underlying factors and mechanisms at play.

## 5. Limitation

Several caveats merit a discussion. Firstly, this study’s distinctive focus on the broader resident population of Naples might pose challenges when comparing the findings with studies conducted in different settings. Nevertheless, this unique perspective can offer unparalleled insights into a phenomenon that involves anyone nowadays. Secondly, the data collected on travel-related infectious disease behaviors relied on self-reported questionnaires, potentially introducing social desirability bias. However, we took steps to reduce this bias by ensuring anonymity and confidentiality for the participants. Thirdly, a potential selection bias could have occurred as certain individuals or groups might have been excluded from our study. Additionally, the analysis may not have accounted for all variables that could influence the outcomes, potentially resulting in a confounding bias. Furthermore, the limited sample size relative to the population being studied limits the generalizability of the findings. Another noteworthy limitation of our study pertains to certain models, particularly Model I, in which we observed a substantial number of statistically significant *p*-values. While a comprehensive and meticulous evaluation of false discovery risks could potentially have contributed to a more precise assessment of alpha error risks, it is important to note that an evaluation of these risks was conducted. The results of this evaluation, including Storey’s *q*-values, were not presented in this paper but were taken into account during our analysis. Lastly, while the Knowledge, Attitudes, and Practices (KAP)-based questionnaire effectively captured insights into travel-related infectious diseases, it may not have encompassed all factors influencing beliefs and behaviors in this context. As a result, future studies should explore additional measures to address this limitation.

## 6. Policies

The findings presented in this study emphasize the critical role of knowledge and attitudes in influencing behaviors related to the prevention of travel-related diseases. It is concerning that many individuals underestimate the risks associated with infectious diseases, indicating gaps in travel health knowledge and preventive behaviors that can lead to hazardous consequences [64]. Given the complexity of travel-related decision-making [65], it is crucial to provide easily accessible and accurate information to empower individuals to make informed decisions about their health and understand the importance and benefits of preventive measures. Enhancing the knowledge of the general population emerges as a valuable strategy as it serves to heighten awareness and enable informed decision-making concerning health behaviors and the prevention of diseases [66]. Healthcare providers also have a vital role in educating individuals about travel-related infectious diseases and preventive measures, including information on vaccinations, medications, and necessary precautions to protect travelers’ health [67]. The importance of raising awareness about travel-related infectious diseases cannot be overstated. This study’s insights serve as valuable guidance for designing evidence-based policies and interventions to tackle the challenges posed by travel-related infectious diseases in the modern era.

## 7. Conclusions

This cross-sectional survey of 1191 adult participants provides insights into the impact of demographic factors, educational attainment, knowledge, attitudes, and behaviors on travel-related infectious diseases. Despite acknowledging design limitations like sampling and social desirability biases, our study’s unique emphasis on the broader resident population of Naples sets it apart from studies solely centered on travelers, offering essential insights for shaping evidence-based policies and public health interventions. This study underscores the significance of multiple factors that impact knowledge, attitudes, and behaviors concerning travel-related infectious diseases. Specifically, knowledge is influenced by age, gender, smoking habits, marital status, and education attainment. Attitudes towards travel-related infectious diseases are shaped by gender, smoking habits, education attainment, marital status, and knowledge. Lastly, behaviors are influenced by marital status, smoking habits, knowledge, and attitudes. Overall, this study highlights the importance of knowledge and attitudes in promoting beneficial behaviors related to travel-related infectious diseases. These findings have major implications for public health policies: raising awareness, targeting the general population, and promoting preventive strategies can help reduce the spread of travel-related infectious diseases, leading to improved public health.

## Figures and Tables

**Table 1:** Study population demographic characteristics.

Study Population	*n*	Percentage
**Sex**	1191	
Male	555	46.6
Female	636	53.4
**Age**		
<30	261	21.9
31–35	123	10.3
36–40	104	8,7
41–45	121	10.2
46–50	101	8.5
>51	481	40.4
**Education**		
Primary school	54	4.5
Middle school	194	16.3
High school	541	45.4
University Degree	402	33.8
**Children**		
Yes	618	51.9
No	573	48.1
**Smoking habits**		
Yes	527	44.3
No	664	55.7
**Marital Status**		
Single	495	41.6
In a relationship	696	58.4

**Table 2:** Results of the multiple linear regression analysis (MLRA).

	Coefficients Not Standardized		Coefficients Standardized			
	b	Standard Error	t	95% Conf. Interval		*p*-Value
**Model I—Dependent variable: Knowledge**						
*Prob > F = 0.000*	*R-squared = 0.4850*			*Root-MSE = 2.8067*		
Age	−0.0238	0.005	−4.68	−0.034	−0.014	0.000
Sex	0.486	0.164	2.97	0.165	0.807	0.003
Marital status	0.578	0.167	3.45	0.250	0.907	0.001
Children	−0.354	0.166	−2.13	−0.680	−0.029	0.033
Smoking habits	0.724	0.166	4.37	0.399	1.05	0.000
Education *						
Middle School	2.46	0.433	5.70	1.62	3.31	0.000
High School	5.60	0.402	13.94	4.81	6.39	0.000
University Degree	8.90	0.408	21.80	8.10	9.70	0.000
**Model II—Dependent variable: Attitudes**						
*Prob > F = 0.000*	*R-squared = 0.4471*			*Root-MSE = 3.1576*		
Age	−0.011	0.006	−1.92	−0.022	0.000	0.055
Sex	0.645	0.185	3.49	0.282	1.01	0.001
Marital status	0.862	0.189	4.55	0.491	1.23	0.000
Children	−0.175	0.187	−0.93	−0.542	0.192	0.35
Smoking habits	0.851	0.188	4.53	0.482	1.22	0.000
Education *						
Middle School	1.16	0.493	2.35	0.192	2.12	0.019
High School	3.95	0.487	8.09	2.99	4.90	0.000
University Degree	4.95	0.543	9.11	3.89	6.02	0.000
Knowledge	0.354	0.033	10.84	0.290	0.419	0.000
**Model III—Dependent variable: Behavior**						
*Prob > F = 0.000*	*R-squared = 0.5703*			*Root-MSE = 4.2197*		
Age	0.009	0.008	1.22	−0.006	0.024	0.223
Sex	0.304	0.248	1.22	−0.183	0.791	0.221
Marital status	0.692	0.255	2.71	0.192	1.19	0.007
Children	−0.636	0.250	−2.54	−1.13	−0.146	0.011
Smoking habits	8.81	2.53	34.81	8.32	9.31	0.000
Education *						
Middle School	−0.350	0.661	−0.53	−1.65	0.947	−0.596
High School	−0.785	0.670	−1.17	−2.10	0.529	0.241
University Degree	−0.272	0.752	−0.36	−1.75	1.20	0.718
Knowledge	0.143	0.046	3.12	0.053	0.233	0.002
Attitude	0.171	0.039	4.40	0.095	0.247	0.000

* Primary school as reference.

## Data Availability

Not applicable.

## Supplementary Materials

The following supporting information can be downloaded at: https://www.mdpi.com/article/10.3390/tropicalmed8100476/s1, Table S1: Knowledge of respondents regarding travel-related infectious diseases; Table S2: Attitude of respondents toward travel-related infectious diseases; Table S3: Behaviors of respondents concerning travel-related infectious diseases.
