# Supplementary material for: Investigating Awareness Regarding Travel-Related Infectious Disease Prevention in a Metropolitan Area
Source: Trop Med Infect Dis. 2023 Oct 18;8(10):476. doi: 10.3390/tropicalmed8100476 (PMC10611143; doi:10.3390/tropicalmed8100476)
Supplement: Supplementary file 1 [file tropicalmed-08-00476-s001.zip › tropicalmed-2659368-supplementary.pdf]

Supplementary Materials

Table S1. Knowledge of respondents regarding travel-related infectious diseases.

N.	Statement *(Variables)*	Agree (%)	Uncertain (%)	Disagree (%)
K1	Infections occur when bacteria, viruses, or fungi enter the body.	46.8	27.9	25.4
K2	A temperature above 38°C is considered a fever.	32.4	35.7	31.9
K3	Contaminated water is a significant source of infection transmission.	40.5	35.1	24.4
K4	Mosquitoes can transmit diseases.	48.3	28.7	23
K5	Infections cannot be transmitted through properly cooked food.	30.9	38.9	30.2
K6	Antibiotics are effective against viruses.	26.7	35.8	37.5
K7	Vaccines stimulate antibody production.	44.9	30.6	24.5
K8	Access to free medical assistance is available in all countries of the world.	25.1	31.6	43.3
K9	Currently, there are 10 mandatory vaccines in Italy.	29.7	46.3	24
K10	Italy's Territorial Units of The Maritime, Air and Border Health Offices (USMAF) provides vaccinations before travel.	25.9	42.3	31.8

Table S2. Attitude of respondents toward travel-related infectious diseases.

N.	Statement *(Variables)*	Agree (%)	Uncertain (%)	Disagree (%)
A1	Packing drugs for travel may not be necessary.	33	31.6	35.4
A2	Disinfecting is necessary after a cut.	44.7	30.1	25.2
A3	Drinking tap water while traveling can be a wiser option.	30.9	32.7	36.4
A4	Covering mouth when sneezing or coughing is not important.	22.7	29.8	47.5
A5	It is important to carry sanitizer.	46.7	29.7	23.6
A6	Eating with hands can be enjoyable.	26.9	32.6	40.5
A7	Mosquito sprays are essentials to have.	37.2	34.4	28.4
A8	Wearing a mask when using public transportation may not be necessary.	36.9	32.9	30.2
A9	Medical insurance is useless when traveling.	29.6	30.2	40.2
A10	It's useful to research necessary vaccines before traveling.	43.2	28.5	28.3

Table S3. Behaviours of respondents concerning travel-related infectious diseases.

N.	Questions	Yes/Always (%)	Often (%)	Sometime (%)	Never (%)
B1	Do you travel abroad?	30.7	27.5	19.6	22.2
B2	Do you carry medications when traveling?	28.3	28.1	13.4	30.2
B3	When you're on vacation, do you try local foods even if they are not cooked?	29.2	27.1	19.1	24.6
B4	Do you only drink bottled water when you're abroad?	27.6	26.9	16.3	29.2
B5	Do you frequently eat food from street vendors when traveling abroad?	27.3	29.5	19.1	24.1
B6	Do you check whether you need any vaccines before going on a trip?	32.2	27.3	15.3	25.2
B7	Do you use products like sprays, diffusers, or bracelets to avoid mosquito bites?	36.9	27.7	14.3	21.1
B8	Do you consume iced drinks often?	34.2	26.7	20.9	18.2
B9	Do you always keep your medical records with you?	29.8	27.7	16.1	26.4
B10	Do you sign up for medical coverage when traveling abroad?	37.3	32.6	12.3	17.8
